# Stroke-Like Symptoms During Sexual Intercourse in a 25-Year-Old Female with a Patent Foramen Ovale

**DOI:** 10.7759/cureus.27332

**Published:** 2022-07-27

**Authors:** Adair M McCabe, Nicholas M Platek, James R Palmieri, Jason R Foerst

**Affiliations:** 1 Family Medicine, Edward Via College of Osteopathic Medicine, Blacksburg, USA; 2 Internal Medicine, Edward Via College of Osteopathic Medicine, Blacksburg, USA; 3 Microbiology and Immunology, Edward Via College of Osteopathic Medicine, Blacksburg, USA; 4 Cardiology, Carilion Clinic, Roanoke, USA

**Keywords:** atrial septal defect, patent foramen ovale, cryptogenic transient ischemic attack, sexual intercourse, bubble study, stroke-like symptoms, amplatzer septal occluder, congenital heart defect

## Abstract

A 25-year-old female who presented with stroke-like symptoms during sexual intercourse was found to have a patent foramen ovale (PFO). She was diagnosed with a cryptogenic transient ischemic attack (TIA) and underwent a successful catheter-based PFO closure. She had complete resolution of symptoms during both intercourse and physical activity.

## Introduction

The septum between the right and left atriums of the embryological heart contains a window, referred to as the foramen ovale, that allows blood to shunt based on the physiologic needs of the fetus [1]. While the closure of this window occurs in most infants by age two, patency remains in over 25% of individuals [1]. Variability in transesophageal echocardiogram (TEE) identifiable characteristics, most notably the size of the patent foramen ovale (PFO) and degree of shunting, influence the risk of clinically significant paradoxical embolic events [2]. A meta-analysis of case-control studies found that PFOs increased the relative risk of stroke in individuals younger than 55 years old by a factor of 3.10 (95% confidence interval (CI), 2.39 - 4.21) [3]. Another prospective study found that the prevalence of PFOs was greater among patients with cryptogenic strokes compared to those with strokes from a known cause (43.9% vs. 14.3%; OR, 4.70; 95% CI, 1.89 to 11.68; P<0.001) [4].

While associations have been made between young adults with PFOs and cryptogenic strokes/transient ischemic attacks (TIAs), there is still insufficient data to draw causation between the two. This case describes a young female who experienced recurring stroke-like symptoms during sexual intercourse and subsequently underwent PFO closure.

## Case presentation

A 25-year-old, otherwise healthy female, presented to her primary care physician complaining of episodes of lightheadedness during sexual intercourse. She reported that these symptoms have always been present during sexual intercourse; however, the most recent episode, approximately five months prior, included dysarthria, numbness of bilateral upper extremities, bilateral vision loss, and unilateral facial drooping lasting approximately five minutes prior to resolution. She also endorsed similar episodes during intense physical activity. The patient denied any history of leg pain, leg swelling, or shortness of breath during any of the episodes or at presentation. The patient had no pertinent medical, surgical, family, or social history. There was no history of smoking or use of estrogen-containing birth control, and her body mass index was appropriate for her height and weight at 22.5. Vitals were all within normal range for her age. Physical examination was unremarkable including a cardiovascular examination that showed normal rate and sinus rhythm with no murmurs, rubs, or gallops and strong palpable distal pulses. Neurological examination revealed intact cranial nerves and no motor or sensory deficits. 

**Figure 1:**
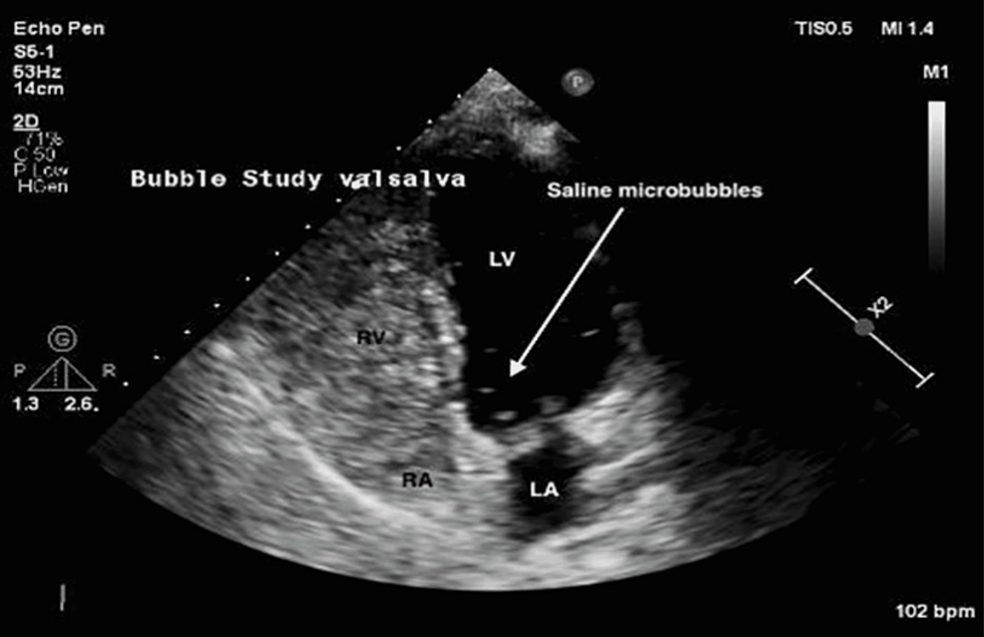
Transthoracic echocardiogram with bubble study pre-procedure. Transthoracic echocardiogram with bubble study during the relaxation phase of the Valsalva maneuver. The microbubbles seen in the left ventricle signify a right-to-left shunt and thus a positive test. Beats per minute (BPM), Left atrium (LA), Left ventricle (LV), Right atrium (RA), Right ventricle (RV).

The patient was examined by cardiology one month later. The initial differential diagnosis included PFO, atrial septal defect (ASD), ventricular septal defect (VSD), vasovagal reflex, and complex migraine. On initial workup, laboratory samples including complete blood count (CBC), complete metabolic panel (CMP), and lipid profile were all within normal limits. Transthoracic echocardiogram (TTE) showed a small PFO with predominantly right to left shunting across the atrial septum and a possible small restrictive membranous VSD. As seen in Figure 1, a bubble study performed at that time was positive. A TEE was obtained for further evaluation and showed a small PFO with no intracardiac thrombi, masses, vegetations, or VSD.

The patient underwent a percutaneous, transcatheter PFO closure approximately one month after the confirmative TEE. Intracardiac echocardiography confirmed the TEE findings of a modest-sized PFO that then easily crossed with a multipurpose catheter. The septum secundum was not thick and there was no atrial septal aneurysm, so a 25 mm Amplatz PFO occluder (Abbott, Santa Clare, CA) was implanted with no residual shunt and normal left atrial pressure. The patient was started on daily 81 mg aspirin and 75 mg clopidogrel.

**Figure 2:**
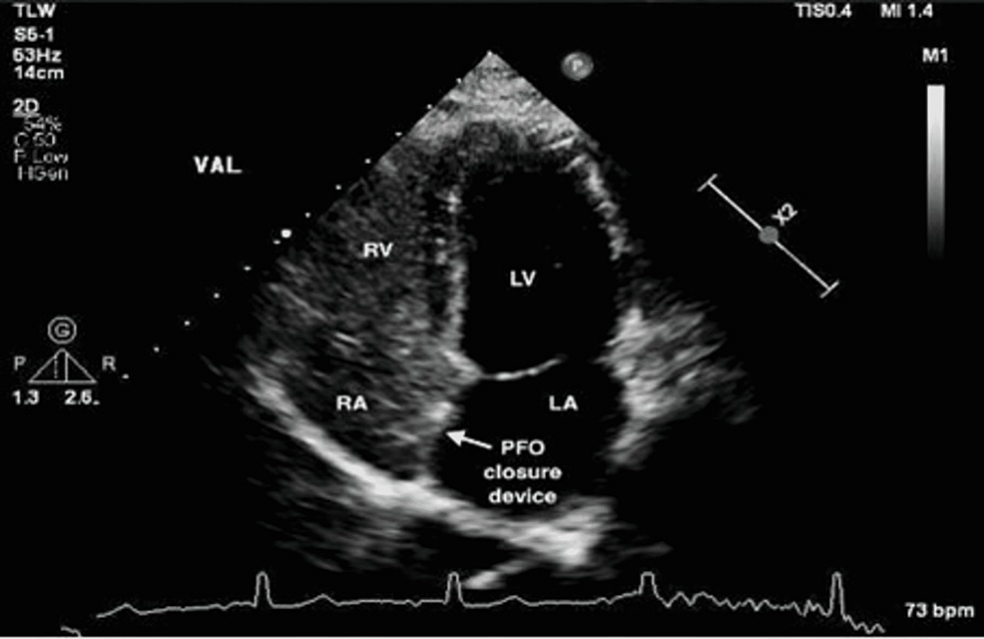
Transthoracic echocardiogram with bubble study post-procedure. Transthoracic echocardiogram and bubble study conducted after the closure procedure showing a negative bubble study and the occluder device in the correct location. Beats per minute (BPM), Left atrium (LA), Left ventricle (LV), Patent foramen ovale (PFO), Right atrium (RA), Right ventricle (RV), Valsalva (VAL).

At her follow-up appointment in the clinic, the patient reported heart palpitations two weeks post-procedure, so a 30-day event recorder was placed. No atrial fibrillation or pauses of three seconds or longer were noted, thus the recorder was discontinued. The patient reported no symptoms during sexual intercourse or physical activity two weeks post-procedure. As seen in Figure 2, a TTE and bubble study were conducted six months post-procedure and showed that the occluder device remained in place with no residual shunting. Use of clopidogrel was discontinued, but the patient remained on aspirin.

## Discussion

There are several studies describing young adult females with a PFO with stroke-like symptoms during sexual intercourse [5]. These patients have insignificant risk factors and an unidentifiable source. One proposed mechanism is an elevation of intrathoracic pressure during sexual intercourse causing a paradoxical embolism through the PFO [5]. Increased intrathoracic pressure causes increased central venous pressure and right atrial pressure, which has the potential to cause a right-to-left shunt. A plausible mechanism to describe the cryptogenic stroke/TIA is an air embolism, which bypasses diagnostic detection yet still causes clinical symptoms. Air embolisms are usually seen during medical procedures like a central line or pacemaker placement, however, there are also reports during sexual intercourse, pregnancy, and in the puerperium [6]. In a report by Moreschi and Broi, potential causes of air embolism linked to sex were “(1) piston-like effect during the penile penetration of the vagina, causing significant mucosal and wall injuries and increasing the quantity of air introduced into endometrial veins which are engorged by sexual arousal; (2) a kneeling position, so that the pelvic region is elevated over the thorax and the right heart level, generating a subatmospheric favorable pressure gradient for the passage of air to peripheral venous vessels; (3) an increase of respiratory ventilation during the consensual intercourse, accentuating the negative intrathoracic pressure during inspiration and causing an important drawing of venous bubbles to the right atrium” [7]. There are also case reports that identify vaginal lacerations as contributors to the formation of air emboli into systemic circulation, although this does not apply to this case report [8]. Another possible explanation is some degree of hyperventilation during intercourse, which has been shown to decrease cerebral blood flow by up to 50% [9]. In addition, several studies have shown that sexual or post-coital headaches were caused by cerebral artery narrowing during or after orgasm [9]. While this patient did not endorse a headache, this mechanism cannot be ruled out.

The question remains as to why this patient experienced classic stroke symptoms but had full resolution of symptoms and no remaining deficits. Gordy and Rowell reported that a lethal volume of air in an acute bolus for humans is theorized to be 3-5 ml/kg (300-500 ml of gas introduced at a rate of 100 ml/sec) but the rate of accumulation and patient position are contributors to lethality [10]. Clinical symptoms of a non-lethal air embolism include dyspnea, continuous coughing, chest pain, seizures, loss of consciousness, altered mental status, and hemiparesis/hemiplegia [11]. This is a plausible explanation for the questions surrounding this patient's case.

Multiple studies have investigated a superior therapy for the prevention of recurrent cryptogenic strokes, including the RESPECT, Gore REDUCE, CLOSE, and DEFENSE-PFO trials. In a recent meta-analysis comparing PFO closure, anticoagulation, and antiplatelet therapy, patients under the age of 60 had a 64% lower risk of stroke recurrence when assigned to the PFO closure group versus the antithrombotic group [12]. Additionally, although there is a low rate of major complications in PFO closure procedures, new-onset atrial fibrillation in closure groups was 4.3 times more frequent than in antithrombotic groups [12,13].

While studies suggest that PFO closure is superior to antithrombotic therapy in a subset of patients, further research is needed in patients with TIAs to look at recurrence, all-cause mortality, and risk versus benefit of procedural complications. The data is not as clear for patients who have experienced TIAs, mostly likely because of the nonspecific symptoms, lack of objective neuroimaging findings, and diagnostic nuances. The lack of pre-closure investigations, such as brain imaging and transcranial Doppler during intercourse, limits our ability to determine the true pathogenesis in this patient and others with similar stroke-like symptoms or TIAs. Future studies that include these tests and investigate mechanisms such as air embolisms may provide answers and lead to early detection and treatment to prevent recurrent symptoms.

## Conclusions

This case demonstrates a unique presentation of stroke-like symptoms during sexual intercourse in a 25-year-old female with a PFO. Further research on PFOs and associated pathological conditions is necessary to explore their contribution to cryptogenic strokes/TIAs. Identifying plausible mechanisms and underlying pathophysiology can help us better understand how to identify patients who are more susceptible to experiencing similar events and subsequently prevent patients from developing serious adverse effects.
